# The Role of Emotions and Motivations in Sport Organizations

**DOI:** 10.3389/fpsyg.2020.00842

**Published:** 2020-05-11

**Authors:** Ana Tur-Porcar, Domingo Ribeiro-Soriano

**Affiliations:** ^1^Faculty of Psychology, Universitat de València, Valencia, Spain; ^2^Institut Universitari d’Investigació en Economia Social, Cooperativisme i Emprenedoria, Universitat de València, Valencia, Spain

**Keywords:** emotions, motivation, sport organizations, commitment, analytic hierarchy process

## Abstract

In sport organizations, a stance aimed at creating a positive emotional and social climate may be necessary. This study examines athletes’ individual psychosocial factors that are linked to sports practice and sports performance. These factors include individual motivation, emotions, and beliefs. The main objective is to create a hierarchy of emotional and motivational factors that sport organizations can use to increase athletes’ commitment. The Analytic Hierarchy Process (AHP) is used to do so. This method enables analysis of priorities and criteria to support decision-making. The results show that motivation, defined here as the drive that leads individuals to develop plans to achieve their goals by balancing short- and long-term goals, and emotion regulation, defined as the capacity to be aware of and manage one’s emotions to reach a balanced emotional state, are the most important criteria to generate this commitment within sport organizations.

## Introduction

Sport organizations play a critical role in the development of sport, especially at an elite level. They do so by not only encouraging athletes’ training but also creating plans to improve athletes’ skills so that they can either achieve excellence or, more broadly, stay fit (Casey et al., 2012; Wagstaff et al., 2012b). Accordingly, a growing body of research analyzes various factors that affect sport organizations in terms of fostering sport and enhancing their quality. Examples of these factors are sports performance (Pain and Harwood, 2008), stress in sports (Kerdijk et al., 2016), organizational success (Weinberg and McDermott, 2002), and organizational citizenship behavior. This organizational citizenship behavior refers to members’ non-formal behavior, which helps the organization to function correctly. Examples include the pressure to participate in unwanted activities and the role of members (Love and Kim, 2019). Nonetheless, research on the factors that influence how sport organizations function is a recent field, and further exploration is needed to understand which factors can improve the internal dynamics of these organizations and help them achieve their fundamental goals.

Sport organizations are social entities that are involved in sport. They have specific objectives and structures to achieve structured, identifiable skills that form part of the sports sector where they operate. Two key elements are the way these organizations function and the management of the organization as a group of individuals working together to achieve the organization’s aims. Hence, good organizational management encompasses the effectiveness, efficiency, and efficacy of relational dynamics (processes), the acquisition of resources (inputs), and the achievement of goals (performance; Wagstaff et al., 2012b).

Therefore, ensuring such organizations function correctly and are well managed partially depends on psychosocial factors that affect the quality of the organization’s internal dynamics, as well as the level of engagement of members in the organization’s goals. So far, however, studies have primarily focused on analyzing individual psychosocial factors that are linked to sports practice and sporting performance, such as individual motivation, emotions, and beliefs (Fletcher and Wagstaff, 2009; Allal-Chérif and Bidan, 2017). In contrast, there is scarce research on the effect of these psychosocial factors on intraorganizational dynamics and, by extension, the commitment of members to creating an ethos of hard work and effort within the organization (Wagstaff et al., 2012b).

It has been shown that factors related to emotions, knowledge of emotions, emotional control, and emotional self-regulation are involved in the way organizations function (Wagstaff et al., 2012b; Didymus and Fletcher, 2017). However, scholars have not yet ranked the importance of knowledge of one’s own emotions and those of others, the use of these emotions, the capacity for emotional self-regulation, and motivation in sport organizations.

In sport organizations, taking measures to create a good emotional and social atmosphere may be necessary. Athletes are immersed in demanding situations. These demands may come from the athletes themselves, the environment, or both. The question is, what factors are necessary to achieve good emotion management in sport organizations?

The analysis in this study was carried out using the Analytic Hierarchy Process (AHP). This structured decision-making technique was used to create an emotion- and motivation-related hierarchy of psychosocial factors based on the judgments of a group of experts in sports practice and organizations. The criteria and variables in relation to emotional control, emotional self-regulation, and motivations were defined on the basis of the literature. They were then presented to the experts, who used their expert opinions to build a hierarchy. In short, this study provides qualitative hierarchical analysis of the importance of knowledge of emotions, emotional self-control, the use of emotions, and motivations in sport organizations.

The rest of the paper is structured as follows. Section “Emotions and Motivation in Sport Organizations” provides a review of the literature on the criteria of emotional self-evaluation and expression, evaluation and knowledge of others’ emotions, the use of emotion, emotional self-regulation, and motivation. Section “Method and Results” describes the method and presents the results. Finally, Section “Discussion and Conclusion” outlines the conclusions and main implications of the study.

## Emotions and Motivation in Sport Organizations

Sport organizations are social entities involved in the sports industry. They have well-defined objectives and structures to achieve structured, identifiable skills that are part of the sports sector where they operate. Therefore, a sports organization is associated with the way an organization functions as a group of individuals who work together to perform tasks that lead to the accomplishment of the organization’s goals (Wagstaff et al., 2012b; Lee and Raschke, 2016).

The correct functioning of an organization partially depends on the psychosocial factors that affect the quality of its internal dynamics and its members’ level of engagement in its goals. Studies have primarily focused on analyzing individual psychosocial factors linked to sports practice and sporting performance, including individual motivation, emotions, and beliefs (Fletcher and Wagstaff, 2009). However, less attention has been paid to how these same factors may influence organizational dynamics and, by extension, the commitment of members to create an ethos of hard work and effort within the organization (Wagstaff et al., 2012b).

Given the importance of establishing a cohesive social environment among the members of sport organizations, the aim of this study is to rank the importance that a group of experts in sports practice and organizations attach to emotional intelligence and individual and collective motivations. The following subsections define the role of one’s own emotions and those of others, the use of emotions, the capacity for emotional self-regulation, and the motivation of individuals in sport organizations.

### Emotional Control and Self-Regulation in Sport Organizations

Studies have highlighted the importance of organizational psychology in sport (Fletcher and Wagstaff, 2009). Sport organizations are groups of people with sport-related objectives who are interested in achieving common goals. Organizations are complex entities. The way they function largely depends on a series of interconnected factors involving the people who make up the organization (Wagstaff et al., 2012a). Accordingly, the internal dynamics of effective sport organizations are based on intra- and inter-personal emotional skills such as the ability to manage one’s own emotions and those of others (Wagstaff et al., 2013). Research by Wagstaff et al. (2012b) identified three emotional skills (identifying, processing, and managing emotions) associated with emotional regulation (i.e., re-evaluation, suppression, and impulse control). These skills are involved in one’s own experiences and relate to the expression of one’s own and others’ emotions.

In the framework of positive psychology, some micro-level changes in people such as positive emotions or fluent relationships between members can extend to the macro level and cause group-level effects (Fredrickson and Dutton, 2008). Therefore, individual factors such as people’s behaviors, emotions, or individual feelings can affect the organization and enhance or hinder its ability to function properly (Wagstaff et al., 2012b). People are involved in this process on an individual level, but this process can also extend to the organization as a whole.

In this theoretical framework, emotional intelligence can be defined as the ability to perceive, understand, express, and manage emotions effectively (Mayer et al., 2004). As such, emotional intelligence involves the knowledge (i.e., the perception and comprehension) of one’s own emotions and those of others, the use of emotions to resolve conflicts effectively, and the regulation or management of one’s own and others’ emotions (Mayer et al., 2016). Accordingly, emotional intelligence enables accurate, well-adjusted thinking about one’s own and others’ emotions and feelings and helps people to think more clearly (Mayer et al., 2008). Emotional knowledge, of both oneself and others, encourages accurate assessment of emotions and supports the management of emotions in stressful situations on an individual and group emotional level. This point is especially relevant in sports (Groves et al., 2008; Lu and Kuo, 2016).

Thus, emotional intelligence can lay the foundations for the skills that play a role in social interactions (Cherniss, 2002). According to Cherniss (2002), the health of a community or organization depends on the effectiveness of the segments of that community or organization, which is made up of people. When these segments do not interact properly, the community or organization suffers, hence the importance of establishing communication channels that are free from emotional tension. The effectiveness of an organization partially depends on the emotional competencies of its members and affects the way the organization functions and is managed.

Furthermore, from an ecological perspective, effective emotional performance depends on the social environment (Hober et al., 2019). In this context, upward spirals (Fredrickson and Joiner, 2002) or positive spirals (Fredrickson and Dutton, 2008) are cycles of improvement in the management of emotions that contribute to building more positive organizations, especially in sports. These positive spirals occur not only upward but also outward from the organization, infusing, and energizing networks and other organizations under this common understanding. Thus, in a sports organization that is sensitive to the emotional control and psychosocial well-being of its members, psychosocial capital will be more readily extended to other members of the organization, which in turn will have more tools for conflict resolution. Fostering an organizational culture of emotional control will influence members’ acceptance of strategies to develop emotional control and skills to build a good rapport with others (Wagstaff et al., 2012b).

In accordance with these theories, the criteria and subcriteria analyzed in this study are emotional self-evaluation and expression, knowledge and evaluation of others’ emotions, the use of emotions, and emotion regulation. In addition to these four criteria, motivation is also considered, as explained later.

*Self-evaluation and emotional expression* (i.e., evaluating and expressing one’s own emotions) is the ability to understand one’s own emotions and to be able to express them naturally (Rieffe and De Rooij, 2012). This criterion is subdivided into three subcriteria: emotional self-assessment, self-understanding of emotions, and self-understanding of feelings. Emotional self-evaluation (i.e., how we value and express our own emotions) is the ability to know how one feels (happy, angry, and sad, etc.), to understand one’s own emotions, and to be able to express these emotions naturally. When expressing anger, fear, anger, sadness, joy, shame, and so forth, a person with this skill is aware of the emotion and uses it adaptively. Anger can be adaptive if it keeps danger at bay; joy is adaptive if it helps us analyze facts with enthusiasm and objectivity; frustration in the face of failure can help change the course of action and encourage future responses, or it can lead to pessimism and the like. A self-understanding of emotions is based on understanding one’s own emotions. It is the ability to analyze emotions and understand them, using objective arguments to know one’s mood if happy, sad, and so on. Finally, a self-understanding of feelings is understanding why one has certain feelings and why one tends to be sad, happy, angry, and so on.

*Appreciation and knowledge of others’ emotions* is the ability to perceive and understand the emotions of other people. This criterion has three subcriteria: the observation of emotions, sensitivity, and understanding of emotions. Observing emotions means being a good observer of others’ emotions and discovering the emotions of others by observing their behavior. The behavior of others is used to interpret their emotions, which involves the ability to observe the emotional state that others are experiencing. Sensitivity means being sensitive to the emotions of others by observing the emotions of others, being able to know their emotions, and putting oneself in the place of others to help with adaptation. Understanding emotions means understanding the emotions of the people nearby. It is the ability to analyze the emotions of others, understand them, and use objective arguments aimed at fully understanding their state of mind to help them.

The *use of emotion* is the ability to use emotions to channel them toward actions that are constructive and allow personal development. This criterion has the subcriteria of mood improvement, self-motivation, and competence. Mood improvement refers to pushing oneself to make the maximum effort and sending positive messages to maintain a mood that is conducive to action. Self-motivation is related to being self-motivated, setting goals, and striving to achieve them. Competence means that people reassure themselves that they are competent. It means trusting one’s self-worth and expressing one’s qualities.

*Emotion regulation* is the ability to be aware of emotions and manage them to reach a balanced emotional state or recover emotionally from situations that cause psychological distress. This criterion consists of emotional awareness and management, emotional self-control, and rationality and temperament. The subcriterion of emotional awareness and management refers to being aware of emotions and handling them in a such a way as to calm down quickly when angry, stay grounded when joyful, keep a cool head in situations of fear, and so on. Emotional self-control means having good control of one’s emotions and being able to control them at all times to prevent one’s emotional state from becoming maladaptive. The subcriterion of rationality and temperament means being able to control one’s temperament and the way one acts to overcome difficulties in a rational, objective manner. For example, temperament can alter the extent to which people are irascible or cheerful and refers to the ability to control that tendency to act more objectively.

### Motivation in Sport Organizations

Research on motivation in sport has primarily focused on the individual dimension, namely the role of motivation in an athlete’s performance or persistence in training (Thøgersen-Ntoumani and Ntoumanis, 2006). However, there seems to be scant research on motivation in the field of sport organizations.

Motivation refers to the psychological processes that orient, energize, and support actions taken toward a goal (Latham and Pinder, 2005). Organizations in general, but specifically sport organizations, that foster an enthusiastic, motivating climate are concerned with the engagement of their members in hard work and effort oriented at improvement. Effort and tenacity are fundamental to support prolonged sporting activity. People in organizations have individual motivations and goals that affect their degree of engagement in work (Escamilla-Fajardo et al., 2016). Therefore, motivation is important in a sports organization.

According to the model of self-determination theory, individuals act proactively in different scenarios and situations of daily life (Deci and Ryan, 2000; Ryan and Deci, 2000). In sports, individuals participate in plans to achieve goals based on causal orientations that may be internal, external, or undetermined. This idea gives rise to the concept of intrinsic versus extrinsic motivation. Intrinsic motivation occurs when an individual does something out of interest or because of the pleasure it produces (i.e., for internal reasons). In contrast, extrinsic motivation is based on the benefits that will be obtained from doing something. Extrinsically motivated individuals participate because of their interest in achieving a goal (Ryan and Deci, 2000). Intrinsic motivation is a form of motivation that is integrated and identified with the individual. It occurs in a process of self-determination whereby individuals focus their efforts until the goal is achieved. It is associated with affective, attitudinal, and behavioral processes (Deci and Ryan, 2000; Deci and Ryan, 2008). People are intrinsically focused on the process. From this perspective, sport organizations that pursue the intrinsic motivation of their components devote their efforts to the process and are supported by affective attitudes of pleasure derived from the work itself (Grant, 2008).

Prosocial motivation is also relevant when efforts to perform tasks are aimed at benefiting others, which is reflected by an empathetic disposition or concern for others (Meglino and Korsgaard, 2004; Grant, 2008). However, as described by Grant (2008), prosocial motivation is linked to intrinsic motivation with some differences. Intrinsic motivation is more process oriented and is oriented toward the present and toward the work itself, which leads to improvement. In contrast, prosocial motivation is oriented toward results, meaning that the outcome of the work will benefit others. Organizations that pursue goals aimed at benefiting others are more focused on the future (Quinn, 2005). Sport organizations have goals related to members’ sports activity. These goals are medium to long term, so they are focused on the present as well as the future. In sport, individuals work for themselves and others. Teamwork is essential in some sports such as football or handball. In this context, the pursuit of benefits to third parties resembles individual objectives, so prosocial motivation may have a strong presence in sport organizations.

**FIGURE 1:**
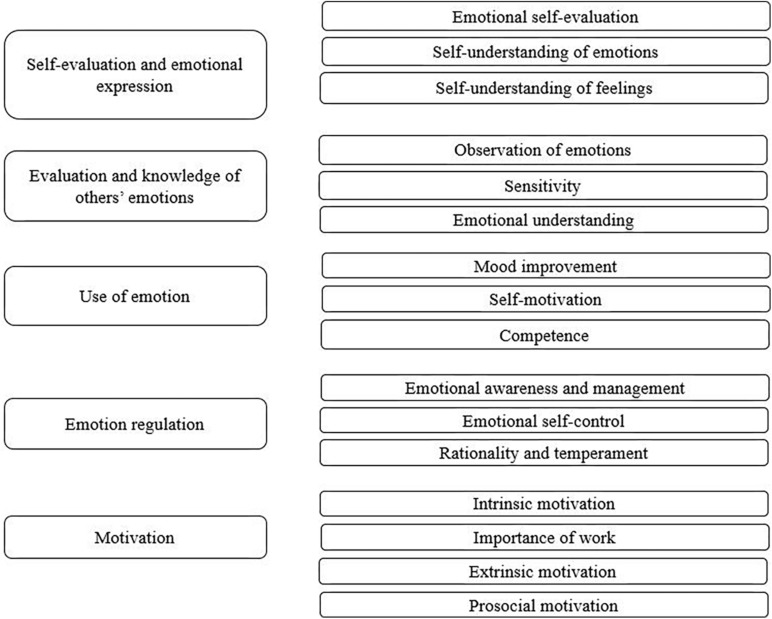
Emotions and motivation within the sports organization.

In addition to the four criteria related to emotional control and self-regulation in sport organizations (i.e., self-evaluation and emotional expression, assessment and knowledge of others’ emotions, use of emotion, and emotion regulation), motivation represents the fifth criterion of the model. Motivation is defined as the drive and energy that leads individuals to create a plan to achieve their goals by balancing short- and long-term objectives. Different types of motivations can be identified, such as intrinsic, extrinsic, and prosocial. Accordingly, the criterion of motivation has four subcriteria: intrinsic motivation, the importance of work, extrinsic motivation, and prosocial motivation. Intrinsic motivation, in reference to engagement with work, alludes to feelings of satisfaction with and enjoyment of work. The importance of work refers to whether the individual adopts persistent attitudes at work to achieve the proposed goals. Extrinsic motivation refers to whether the results of the work yield financial or social benefits or benefits in the form of recognition. Extrinsic motivation means that work is seen as important if it provides the rewards that individuals want to achieve, working only for external incentives. Finally, prosocial motivation means that the task is carried out thinking of the benefit it will have for others, where the individual performing the task has other people in mind. The work is designed to benefit and help others. Altruism is part of this type of motivation. Based on these criteria, the model analyzed using the AHP is presented in Figure 1.

## Method and Results

The AHP enables analysis of priorities and criteria to support decision making. The AHP was developed in the 1970s by Thomas Saaty, who used mathematical and psychological knowledge to build this method. The AHP identifies the best alternative in highly uncertain contexts (Soma, 2003).

The AHP requires the identification of the criteria that will form the hierarchy. The AHP focuses on two aspects: hierarchical design and evaluation (Vargas, 1990). These criteria can be identified from, for example, a review of the literature, a pilot survey, or interviews with experts. The hierarchy lets researchers group the criteria into categories such as social problems, educational questions, political subjects, sustainability issues, and the like. The upper levels of the hierarchy represent the general objectives, and the lower levels relate to specific criteria and subcriteria, which represent the alternatives to be selected. This study adopts the comparative scale defined by Saaty (1987, 1990, 2008) to determine the preference of each criterion.

To identify and sort the preferences, pairwise comparison of the criteria is used. Each comparison is assigned a value to indicate the extent to which one alternative is preferable to another. The scale varies from 1 to 9, where 1 indicates that the criteria are equally important and 9 indicates that the importance of one of the elements is much greater than the importance of the other. The aim of this process is to establish the relationships of preference between pairs of criteria from the top level. Once the criteria have been compared, the consistency of the judgments must be verified. Finally, the judgments are combined and analyzed to yield the priorities and ranking (Borade et al., 2013).

The AHP has been used extensively in research in recent years. Although it has been applied to multiple fields, it is mainly used in management decision-making problems (Aragonés-Beltrán et al., 2017) and in studies of environmental problems (Shekhar and Pandey, 2015; Yuan et al., 2015).

Emotion-related factors as well as knowledge of emotions, emotional control, and emotional self-regulation are involved in the way an organization functions (Wagstaff et al., 2012b; Didymus and Fletcher, 2017). In this case, it is a question of observing their importance within sport organizations to rank the emotion-related criteria using the AHP. For this purpose, the criteria that were used in this study are shown in Figure 1. As per the method employed by Castrogiovanni et al. (2016), Mas-Tur et al. (2019), and Roig-Tierno et al. (2013), a group of seven experts from sport organizations was selected, that were in the direction and management of sports organizations or were elite athlete or long-term users. In average, the experts were connected with sports organizations over 10 years. Four were men, and three were women.

Table 1 shows the main results of the study. The most important factor (criterion) to explain commitment within sport organizations is motivation, with a rating of 40.3%. In other words, the drive that leads people to create a plan to achieve goals by balancing short- and long-term aims is the most important criterion for feeling commitment within sport organizations. The next criterion relates to emotional regulation and has a weight of 25.8%. Together with motivation, people’s ability to be aware of their emotions and manage these emotions to reach a balanced emotional state and recover emotionally from situations of psychological distress is particularly important in sport organizations.

**TABLE 1:** Ranking of criteria and subcriteria.

			Relative	Total
			weight	weight
Emotional self-evaluation	8.9%	Self-assessment	42.3%	3.8%
		Understanding emotions	19.3%	1.7%
		Understanding feelings	38.4%	3.4%
Others’ emotions	10.3%	Observation of emotions	15.1%	1.6%
		Sensitivity	54.8%	5.6%
		Understanding	30.1%	3.1%
Use of emotions	14.7%	Improving mood	23.3%	3.4%
		Self-motivation	38.0%	5.6%
		Competence	38.7%	5.7%
Regulation	25.8%	Emotion management	40.8%	10.5%
		Self-control	37.4%	9.7%
		Temperament	21.8%	5.6%
Motivation	40.3%	Intrinsic	47.1%	19.0%
		Work	19.3%	7.8%
		Extrinsic	8.4%	3.4%
		Prosocial	25.2%	10.2%

Secondly, the results show the most important subcriterion within each criterion. Two subcriteria are particularly important: intrinsic motivation (i.e., involvement in sport combined with satisfaction and enjoyment of work), with a score of 19.0%, and management of emotions (i.e., being aware of emotions and handling them to keep a cool head in situations of fear, stress, and the like), with a weight of 10.50%.

Table 2 shows the ranking of the subcriteria. The results show that 16 subcriteria explain the commitment of individuals within sport organizations. The first four subcriteria in Table 2 account for almost 50% of the total weight of all criteria.

**TABLE 2:** Ranking of subcriteria.

Ranking	Subcriteria	Total weight	Sum
1	Intrinsic	19.0%	49.4%
2	Management	10.5%	
3	Prosocial	10.2%	
4	Self-control	9.7%	
5	Work	7.8%	
6	Competence	5.7%	
7	Sensitivity	5.6%	
8	Self-motivation	5.6%	
9	Temperament	5.6%	
10	Self-assessment	3.8%	
11	Understanding feelings	3.4%	
12	Improving mood	3.4%	
13	Extrinsic	3.4%	
14	Understanding	3.1%	
15	Understanding emotions	1.7%	
16	Observation	1.6%	

Notably, of all the subcriteria, the four with the highest percentage weight relate to emotional regulation and motivation. These two criteria, as mentioned previously, must be emphasized in reference to increasing the individuals’ commitment to sports.

In addition, the subcriteria related to evaluating one’s own emotions and those of others have little importance in relation to the other subcriteria. This result implies that, according to the experts, these criteria have no further relevance when considering commitment within sport organizations.

## Discussion and Conclusion

This study examined the criteria that are most affected by the commitment of athletes to sport organizations. For this purpose, the AHP (Saaty, 1990, 2008) was used to indicate the experts’ preference for each criterion. Vaidya and Kumar (2006) reported that the AHP has been applied in a number of scientific areas, including education, politics, and industry. In addition, the number of applications of the AHP has increased in the areas of organizational management, knowledge, and entrepreneurship in recent years (Roig-Tierno et al., 2013; Castrogiovanni et al., 2016; Tur-Porcar et al., 2018; Mas-Tur et al., 2019).

### Theoretical and Practical Implications

The results have several implications. First, motivation, especially intrinsic motivation, and seems to be fundamental in sport organizations. The experts indicate that organizations must be sensitive to foster the intrinsic motivation of their members to boost athletes’ training, engage athletes in improving sports skills, and achieve a positive emotional and social climate (Casey et al., 2012; Wagstaff et al., 2012b). People who are committed to sport and who gain a sense of satisfaction from doing sport have an internal drive. Therefore, the strength to continue doing sport comes from within. They embark on a process of self-determination, improvement, and orientation of their activity, which has positive consequences for sport organizations (Deci and Ryan, 2000; Deci and Ryan, 2008).

Furthermore, in sport organizations, prosocial motivation is also highly accepted by experts. In team sports, individual effort makes no sense if it does not benefit others. In sport, it is essential to think of the benefit to others and not only one’s own benefit. The ability to think of others and be empathetic by showing concern for others is an important aspect in sport organizations (Meglino and Korsgaard, 2004; Grant, 2008). Furthermore, prosocial motivation is linked to intrinsic motivation in such a way that it is difficult for prosocial motivation to exist without intrinsic motivation (Grant, 2008), although each form of motivation has its own nuances.

Also on the theme of motivation, the results show that extrinsic motivation plays only a minor role. Sport and the internal atmosphere of sport organizations depend only slightly on this type of motivation. The contribution of external motivations such as financial incentives or winning competitions is of relatively little importance in the experts’ opinion. However, the different forms of motivation might encourage one another (Deci and Ryan, 2000; Grant, 2008). One example of this is when efforts made to improve well-being are accompanied by excellent team results and a competitive victory.

Second, in addition to motivation, emotion regulation is an especially relevant criterion. Emotion regulation is the capacity of the members of the sports organization to know and manage their emotions effectively (Wagstaff et al., 2012b; Didymus and Fletcher, 2017). Identifying, processing, and managing one’s own as well as others’ emotions effectively can help members of sport organizations control impulses in conflictive or stressful situations (Wagstaff et al., 2012b). An environment where most members display emotional regulation is more likely to spread to the rest of the members of the organization, thereby creating a social climate that is conducive to emotional control and regulation in the macro-functional dimension (Fredrickson and Dutton, 2008; Wagstaff et al., 2012b).

Thus, sport organizations that are able to instill the management of emotion in their members can deal more effectively with stressful situations, which frequently arise in sport (Groves et al., 2008; Kerdijk et al., 2016; Lu and Kuo, 2016), either to pursue excellence or to promote good health (Casey et al., 2012; Wagstaff et al., 2012b).

Third, the self-evaluation of one’s own emotions and those of others is of little importance in the functioning of sport organizations. Although emotional intelligence enables more suitable thoughts about one’s own and others’ emotions and feelings, which aids clearer thinking (Mayer et al., 2008), it seems that this criterion is not relevant to the way sport organizations function. Nevertheless, emotional knowledge of oneself and others is conducive to the management of emotions in stressful situations, which is especially important in sport (Groves et al., 2008; Lu and Kuo, 2016).

### Research Limitations and Directions for Future Research

This study has some limitations. First, it is based on experts’ opinions regarding certain criteria. The opinion of these experts may have biases because of their own sports background or the specific sport being considered. This limitation offers an opportunity for future research based on determining which criteria are most relevant in different sports disciplines. This weakness can just as well be viewed from another angle as a strength because the method is based on the judgments of experienced people. This basis makes this research relevant as a preliminary work on which to develop the theme of emotions as key factors of commitment in organizations. This issue has not yet been studied, despite its growing importance (Wagstaff et al., 2012b; Didymus and Fletcher, 2017). Another limitation derives from the definition of the terms. These linguistic terms are subject to changes and nuances in their definitions. They were therefore defined and presented in the questionnaire so that the respondents had a clear definition of each criterion and subcriterion before responding.

## Data Availability Statement

The original contributions presented in the study are included in the article/supplementary material, further inquiries can be directed to the corresponding author.

## Author Contributions

AT-P and DR-S designed and performed the experiments, analyzed the data and wrote the manuscript.

## Conflict of Interest

The authors declare that the research was conducted in the absence of any commercial or financial relationships that could be construed as a potential conflict of interest.
